# Effectiveness of Artificial Intelligence–Assisted Decision-making to Improve Vulnerable Women’s Participation in Cervical Cancer Screening in France: Protocol for a Cluster Randomized Controlled Trial (AppDate-You)

**DOI:** 10.2196/39288

**Published:** 2022-08-02

**Authors:** Farida Selmouni, Marine Guy, Richard Muwonge, Abdelhak Nassiri, Eric Lucas, Partha Basu, Catherine Sauvaget

**Affiliations:** 1 Early Detection, Prevention & Infections Branch International Agency for Research on Cancer Lyon France; 2 Regional Cancer Screening Coordinating Centre of Occitanie Carcassonne France; 3 Faculty of Law, Economics, Management and Economic and Social Administration University of Western Brittany Brest France

**Keywords:** cervical cancer, screening, chatbot, decision aid, artificial intelligence, cluster randomized controlled trial

## Abstract

**Background:**

The French organized population-based cervical cancer screening (CCS) program transitioned from a cytology-based to a human papillomavirus (HPV)–based screening strategy in August 2020. HPV testing is offered every 5 years, starting at the age of 30 years. In the new program, women are invited to undergo an HPV test at a gynecologist’s, primary care physician’s, or midwife’s office, a private clinic or health center, family planning center, or hospital. HPV self-sampling (HPVss) was also made available as an additional approach. However, French studies reported that less than 20% of noncompliant women performed vaginal self-sampling when a kit was sent to their home. Women with lower income and educational levels participate less in CCS. Lack of information about the disease and the benefits of CCS were reported as one of the major barriers among noncompliant women. This barrier could be addressed by overcoming disparities in HPV- and cervical cancer–related knowledge and perceptions about CCS.

**Objective:**

This study aimed to assess the effectiveness of a chatbot-based decision aid to improve women’s participation in the HPVss detection-based CCS care pathway.

**Methods:**

AppDate-You is a 2-arm cluster randomized controlled trial (cRCT) nested within the French organized CCS program. Eligible women are those aged 30-65 years who have not been screened for CC for more than 4 years and live in the disadvantaged clusters in the Occitanie Region, France. In total, 32 clusters will be allocated to the intervention and control arms, 16 in each arm (approximately 4000 women). Eligible women living in randomly selected disadvantaged clusters will be identified using the Regional Cancer Screening Coordinating Centre of Occitanie (CRCDC-OC) database. Women in the experimental group will receive screening reminder letters and HPVss kits, combined with access to a chatbot-based decision aid tailored to women with lower education attainment. Women in the control group will receive the reminder letters and HPVss kits (standard of care). The CRCDC-OC database will be used to check trial progress and assess the intervention’s impact. The trial has 2 primary outcomes: (1) the proportion of screening participation within 12 months among women recalled for CCS and (2) the proportion of HPVss-positive women who are “well-managed” as stipulated in the French guidelines.

**Results:**

To date, the AppDate-You study group is preparing and developing the chatbot-based decision aid (intervention). The cRCT will be conducted once the decision aid has been completed and validated. Recruitment of women is expected to begin in January 2023.

**Conclusions:**

This study is the first to evaluate the impact of a chatbot-based decision aid to promote the CCS program and increase its performance. The study results will inform policy makers and health professionals as well as the research community.

**Trial Registration:**

ClinicalTrials.gov NCT05286034; https://clinicaltrials.gov/ct2/show/NCT05286034

**International Registered Report Identifier (IRRID):**

PRR1-10.2196/39288

## Introduction

### Background

In France, there were an estimated 2920 new cervical cancer (CC) cases and 1117 related deaths in 2018, with marked geographical disparities within regions [1]. A decline in CC incidence and mortality rates was observed during the period of 1990 to 2018, although this was less pronounced in recent years [1]. The prognosis of CC is deteriorating in France, with a 5-year survival rate after diagnosis decreasing from 68% in 1989 to 1993 to 62% in 2005 to 2010 [2]. During the period of 2018 to 2020, the national triennial coverage rate was 59.2%, with a strong geographical disparity within French departments, ranging from 11.8% to 65% [3]. The national screening coverage rate appeared relatively stable since 2012 across the age groups and declined markedly in women older than 50 years, down to 44.2% in women aged 60 to 65 years [4].

The French organized population-based CC screening (CCS) program shifted from a cytology-based to a human papillomavirus (HPV)–based screening strategy in August 2020. HPV testing is offered every 5 years, starting at the age of 30 years. In the new program, women are invited to have the HPV test at a gynecologist’s, primary care physician’s, or midwife’s office, a private clinic or health center, family planning center, or hospital. A reminder letter is sent to noncompliant women after 12 months from the date of the first screening invitation.

HPV self-sampling (HPVss) was also made available as an additional approach to recall noncompliant women 1 year after the initial invitation [5]. There is a strong body of evidence to support the usefulness of HPVss in increasing participation of hard-to-reach women in screening programs [6,7]. In France, 2 randomized controlled trials (RCTs) have been conducted to evaluate home-mailing of HPVss kits directly to noncompliant women in 2 regions [8,9]. Home-based HPVss testing was accepted among French women and increased participation in CCS compared with a recall letter: 18.3% versus 2.0% [8] and 22.5% versus 11.7% [9], respectively, among nonattendees. Some concerns remain regarding adherence to further follow-up among high-risk women with positive test results. The French studies reported substantial variation in compliance with follow-up among HPVss-positive women (41% vs 92%) [8,9]. These studies also found that less than 20% of noncompliant women performed vaginal self-sampling when an HPVss kit was sent to their home. Women are concerned about the self-test’s effectiveness and are afraid of hurting themselves when collecting the sample [10].

In France, women older than 50 years, those with unfavorable socioeconomic status, those living in disadvantaged and in low-medical-density areas, those with long-term disease, and those covered by *complementary health insurance* are more likely to not participate in CCS [11]. Lack of information about CC and the benefits of CCS were reported as two of the major barriers among noncompliant women [12-14]. These barriers could be addressed by overcoming disparities in HPV- and CC-related knowledge and perceptions about CCS.

Studies showed that decision aids improve knowledge, decrease decisional conflict, and increase the proportion of individuals who are active in the decision-making process [15,16]. Decision aids must be carefully established, tested and validated by users, and meet their needs [17]. Standard information materials, even with simple text and design, are not an appropriate communication tool for individuals with low health literacy [18]. In contrast, animated educational videos were found to be the best way to communicate complex health information to such individuals [19]. Health literacy is defined as “[people’s ability] to make judgements and take decisions in everyday life concerning healthcare, disease prevention and health promotion to maintain or improve their quality of life” [20]. Low health literacy is prevalent among people who are older, less educated, low-income, have chronic conditions, and do not speak the native language of the country where they live [21]. In France, the prevalence of low health literacy was estimated to be 51% (95% CI 34%-67%) [22].

One of the useful innovative tools being used to self-administer web-based health information is the chatbot. Chatbots, as artificial intelligence (AI) devices, are applications that provide information or services through interactions with users [23]. To our knowledge, no study has evaluated the usefulness of AI-based chatbot services to empower women and democratize the decision-making process about CCS. This study will be the first to develop and test a chatbot-based decision aid tailored to women with lower education attainment. Previous systematic reviews have shown that no RCTs have examined shared decision-making in CCS programs [16,24]. This paper describes the objectives and protocol of the cluster randomized trial (cRCT) using the Standard Protocol Items: Recommendations for Interventional Trials–Patient-Reported Outcomes (SPIRIT-PRO) guidelines.

### Objectives and Endpoints

The primary objective of this AppDate-You study is to assess the effectiveness of a chatbot-based decision aid to improve women’s participation in the HPVss detection–based CCS care pathway, in particular among noncompliant women living in disadvantaged areas in Occitanie Region, France.

The main secondary objectives are to evaluate the impact of the intervention on (1) the detection rate of cervical intraepithelial neoplasia grade 2+, (2) the intervals between the dates of the reminder letter and the HPVss test report and between the dates of sending a positive HPV test result and performing liquid-based cervical cytology (triage test), and (3) the efficiency (cost-effectiveness) of the intervention.

## Methods

### Study Design

**Figure 1:**
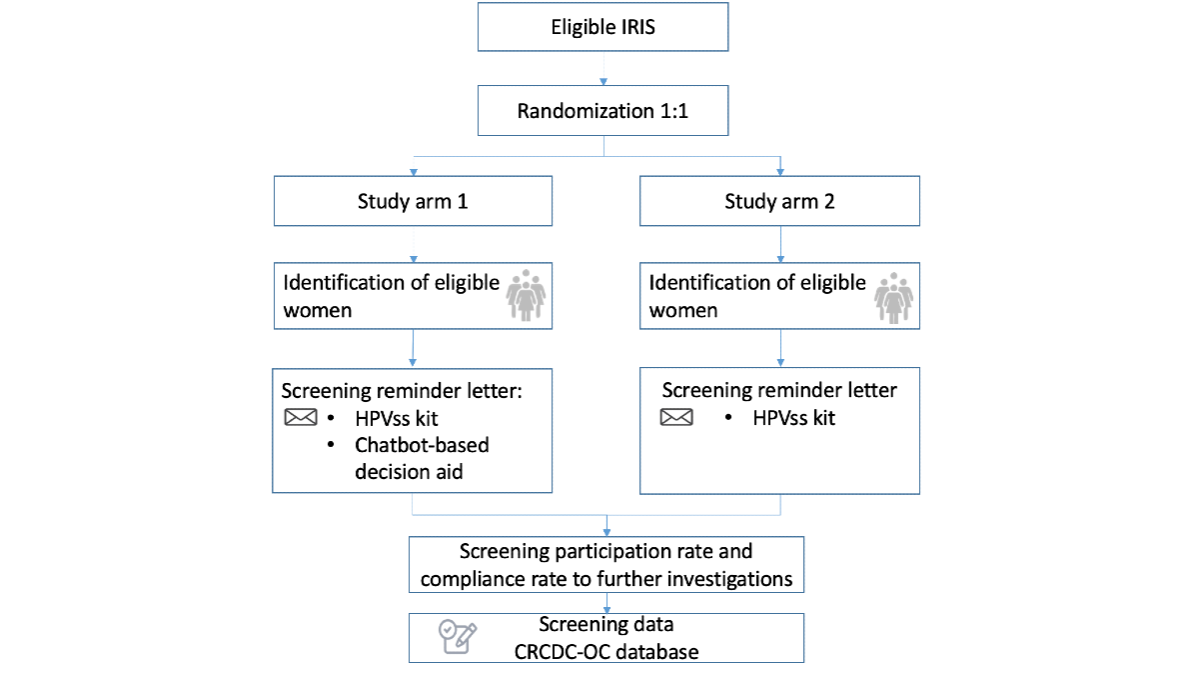
Flowchart of the study. CRCDC-OC: Regional Cancer Screening Coordinating Centre of Occitanie; HPVss: human papillomavirus self-sampling; IRIS: Ilots Regroupés pour l’Information Statistique.

A 2-arm cRCT will be conducted (Figure 1). A cluster is defined by aggregated units for statistical information (*Ilots Regroupés pour l’Information Statistique;* IRIS), corresponding to 2000 inhabitants per unit. Only IRIS classified as 4 or 5 (the most disadvantaged IRIS) in accordance with the French version of the European Deprivation Index will be included [25]. The study will be nested within the French CCS program in the Occitanie Region. Recruitment of women will start in January 2023 and end in December 2024, and HPVss-positive women will be followed-up for 12 months after the last participants are recruited.

### Study Intervention

**Figure 2:**
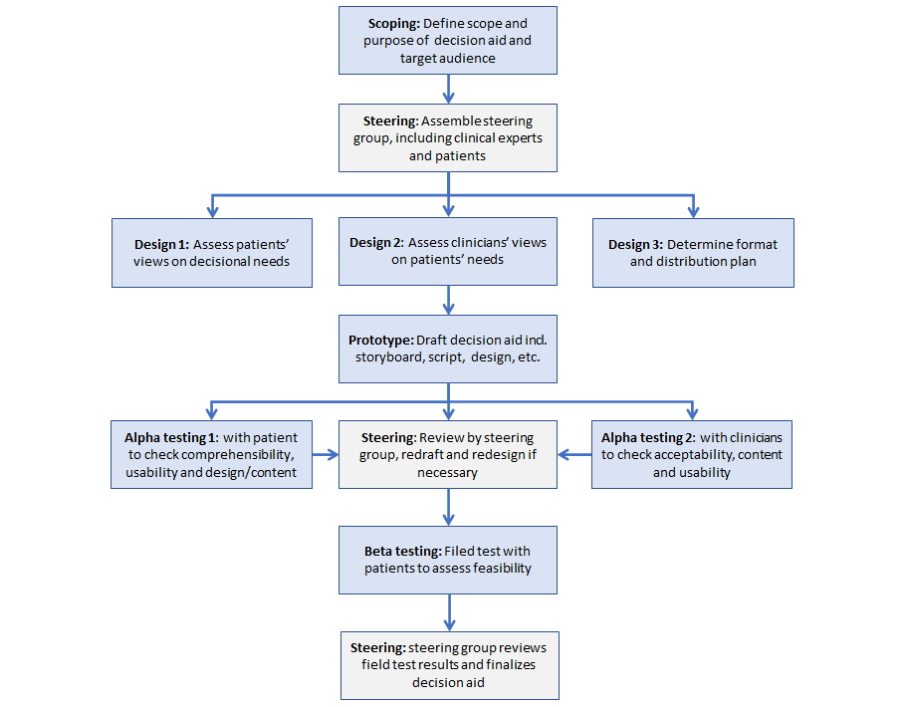
Model development process for decision aids (adapted from Coulter et al [17]).

The chatbot-based decision aid will be developed using the validated Coulter framework on the basis of the International Patient Decision Aid Standards (IPDAS) [17]. This framework proposes 5 steps (Figure 2): (1) scoping and design, (2) development of a prototype, (3) “alpha” testing with women and clinicians in an iterative process, (4) “beta” testing in “real-life” conditions (field tests), and (5) production of a final version. The chatbot-based decision aid will be tailored to women with a lower education level and will be offered in several languages (the most spoken languages in the Occitanie region) and via several channels.

### Study Population

Eligible women are those aged 30-65 years who did not respond to an initial invitation letter to have an HPV test collected by a clinician, have not been screened for CC for more than 4 years, and live in the disadvantaged clusters in the Occitanie Region.

Women will be excluded from the study if they have undergone CC screening within the past 4 years, have had a hysterectomy, or have been diagnosed with and are receiving treatment for precancer or CC.

### Sample Size

The sample size involved estimation of the number of clusters (IRIS) using a composite outcome, “well-managed” women, which is defined as those women who completed a valid HPVss test and were either advised of a negative result (in the case of HPVss-negative women) or completed the assessment (in the case of HPVss-positive women), and, where necessary, treatment pathway. The following assumptions were considered:

- Expected average cluster size of 250 eligible women; this is an estimate of the number of women in the target age range (30-65 years) within a cluster of 2000 people, based on the population pyramid of ages in the Occitanie Region.
- Expected proportion of overall well-managed women in the control group of 15%.
- Expected absolute increase of 10% in the proportion of overall well-managed women in the experimental group.
- Coefficient of variation taking into account the possibility of varying cluster sizes of 1.2 (arrived at because of the wide level of dispersion around the mean cluster size) [26].
- A 10% precision in the proportion of overall well-managed women, which would lead to a 0.029 intracluster correlation using a beta-binomial model [27].
- A power of 80% and a 5% 2-tailed significance level.

Under the aforementioned assumptions, the required number of clusters to randomize in each arm would be 10 (total: 20 clusters). However, if subgroup analyses are to be performed, stratifying on general population density, medical density of the department (high or low), and setting of the community (rural or urban), taking into consideration these α adjustments (α=.3125% [2.5%/8]) for each of the subgroup analyses in order to be able to remain with a 5% 2-tailed significance level for the overall effect, approximately 16 clusters in each arm (32 clusters in total) will be required for the subgroup analyses.

### Randomization

A sampling frame of a list of disadvantaged clusters was obtained from Caen University, Caen, France. Clusters (IRIS) will be randomly assigned to the 2 randomization groups in a 1:1 ratio using computer-generated simple randomization. Eligible women living in the randomly selected disadvantaged clusters will be identified using the Regional Cancer Screening Coordinating Centre of Occitanie (CRCDC-OC) database.

### Study Procedures

Eligible women in both groups will receive screening reminder letters and HPVss kits. The HPVss kit will contain a self-adhesive barcode, an HPVss kit, instructions with illustrations on how to self-collect the vaginal samples, and a postage-paid, preaddressed return envelope. Women in the experimental group will receive an information letter explaining the nature of the research, its objective, and what participants will be required to do and how to access the chatbot-based decision aid.

Participants in both groups will be requested to either perform vaginal self-sampling at their home (with the collection kit provided) or have an HPV test with their gynecologist, primary care physician, or midwife. Those who choose HPVss will be encouraged to contact the CRCDC-OC via telephone or email in the event of any uncertainty.

### HPV Test Analysis and Management of Results

Participating women in both groups will be asked to send the samples via regular mail to a centralized laboratory using the postage-paid envelope. The results will be communicated to the women, their primary care physician, and the CRCDC-OC generally within 2 weeks after the self-collected sample arrives at the centralized laboratory. Women with negative results will be advised to repeat CCS after 5 years. HPVss-positive women will be invited to consult their primary health physician, who will explain what a positive HPVss test result means and suggest further investigations in accordance with national guidelines [28].

HPVss-positive women will be referred for liquid-based cervical cytology triage:

- HPVss-positive women with a cytological diagnosis of atypical squamous cells of undetermined significance or worse will be referred for colposcopy.
- HPVss-positive women with a normal cytological diagnosis will be advised to undergo HPV testing 1 year later. If this triage HPV test, performed 1 year later, yields positive results, colposcopy should be performed; if this triage HPV test yields negative results, another HPV test should be offered 5 years later.

For women with an uninterpretable HPV test, a new HPVss collection kit will be sent to them. If the second HPV test result is also uninterpretable, the women will be advised by mail to have an HPV test with a health professional as soon as possible.

The CRCDC-OC will track the HPVss-positive women to investigate whether they receive follow-up procedures in accordance with national recommendations and will recall women or providers, if necessary.

### Data Collection and Study Variables

The study data will be generated from the CRCDC-OC database. The source of the CRCDC-OC database is the French health insurance database (*Système National des Données de Santé*; SNDS). This database contains individualized and deidentified data on all medical expenditures and reimbursements. All medical care received in the public, private, or liberal sector is recorded in the SNDS. No additional personal or clinical information will be collected during this trial. Anonymized data for each individual participant will be received, stored, and handled at International Agency for Research on Cancer (IARC) using Research Electronic Data Capture, a secure, web-based software platform designed to support data capture for research studies [29,30]. All standard precautions will be taken to ensure the privacy and protection of personal and medical information. Only the principal investigators and coinvestigators as well as the data manager will have access to the data.

This database enables access to some data on women’s characteristics (date of birth, place of residence, and type of health insurance), screening (HPV testing), and further investigations (cytology, colposcopy, biopsy, treatment of precancerous lesions, and surgery).

Real-time usage statistics generated by the chatbot platforms will be used to evaluate women’s use of the chatbot. Chat volumes, response time to women’s chat requests, the number of queries resolved, the main topics in women’s requests, navigation flow, and women’s satisfaction will be analyzed.

### Primary Study Outcomes

The study has 2 primary outcomes: (1) the proportion of screening participation within 12 months among women recalled for CCS and (2) the proportion of HPVss-positive women who are “well-managed” as stipulated in the French guidelines. Both proportions will be compared between experimental and control groups, taking into account the stratification by age group, place of residence, and type of health insurance.

### Secondary Study Outcomes

We will examine whether the intervention also improves the proportion of women providing valid vaginal samples and the proportion of women with invalid HPVss tests who repeat HPVss. We will also compare the detection rate of cervical intraepithelial neoplasia grade 2+ in the 2 groups. Median intervals between the date of the reminder letter and the date of the HPVss test report, and between the dates of sending a positive HPV test result and performing liquid-based cytology will be compared between groups, stratified by age group, place of residence, and health insurance type.

An evaluation of cost-effectiveness will also be performed, to first estimate the cost of the intervention and to calculate the incremental cost-effectiveness ratios as the mean difference in total costs between the intervention and control groups with the mean difference in effects, and expressed as both Euros and US dollars per percentage change in screening participation. Deterministic and probabilistic sensitivity analyses will also be conducted by varying key parameters that may affect the outcome and conclusions of the economic evaluation.

Women’s use of the chatbot will also be assessed through usage and engagement statistics generated by chatbot platforms. We will estimate the average number of messages each participating woman exchanged with the chatbot, the average interaction time spent by each participant and a session-wise split of duration, the average number of failed messages that the Chatbot failed to respond, the most frequently asked questions, and the language most often used to seek information, etc.

### Statistical Analysis

The participants’ categorical characteristics will be presented as proportions compared between the intervention and control groups using chi-square analysis. Continuous variables will be presented as median (interquartile range) values. Comparisons will be carried out using Kruskal-Wallis tests. The effect of the intervention on both the primary and secondary outcomes will be assessed using logistic regression models, adjusting for individual characteristics that are statistically significant in the univariate analysis and the cluster design. The effect estimates obtained from the regression analysis will be presented as odds ratios together with their 95% CIs. Just Another Gibbs Sampler software will also be used to model missing data in outcomes and/or explanatory variables [31].

### Ethics Approval

The study protocol will be submitted to the French and the IARC ethics committees once the chatbot-based decision aid has been completed. No individual consent will be required; all participants will be informed of their rights not to participate or to object to the collection of their data. The design phase of the chatbot-based decision aid was approved by the IARC ethics committee (IEC 21-16).

### Study Organization

The project will be coordinated by 2 committees. The steering committee (the AppDate-You Study Group) includes all scientific leaders and their collaborators of the 3 study teams: at the CRCDC-OC, the University of Western Brittany, and the IARC. This committee meets on a monthly basis to discuss all scientific and organizational aspects of the project and to decide on corrective measures to be taken, if necessary.

The scientific committee is composed of experts (an epidemiologist, a public health specialist, a gynecologist, and a sociologist), who are independent of the trial, and the scientific leaders of the 3 study teams. This committee meets once a year to make recommendations on the progress of the trial.

## Results

To date, the AppDate-You study group is preparing and developing the chatbot-based decision aid (intervention). Once the decision aid has been completed and validated, we will conduct the cRCT as described above. Recruitment of women is expected to begin in January 2023. The results of the study will be the subject of a new academic publication.

## Discussion

The HPVss test was recently introduced into the French organized CCS program for women who do not participate in regular screening. The goal of our intervention is to improve the effectiveness of the HPVss strategy among women living in deprived areas. If the proposed intervention is successful, the findings of this trial will inform policy makers and stakeholders to update local guidelines. The intervention would be easy to integrate into the current screening program and expand to other populations. Information tailored to audiences with low health literacy is also appropriate for people with high health literacy [19]. A chatbot-based decision aid solution can also be used in other screening or health programs.

There is vast interest in how to optimize the send/return ratio of HPVss kits. We expect our intervention tool to be accepted by women, with a large impact on women’s participation in CCS. First, this is a validated framework based on a collaborative decision-making process among women, health professionals, health psychologists, decision-making experts, and the AppDate-You Study Group and a pilot study to ensure the feasibility and acceptability among the targeted women. Second, the COVID-19 pandemic has accelerated the adoption of digital uses; the French government and citizens turned toward digital technologies to respond to the health crisis and address a wide range of pandemic-related issues. According to the 2021 Digital Barometer, 94% of respondents in France use e-banking (+ 1 point from 2020), 93% have adopted e-administration (same as in 2020), 91% use e-commerce (+ 3 points), 87% use social networks (+2 points), and 83% use instant messaging (+ 1 point) [32]. Services using AI are up by 6 points and are used by 70% of French people [32].

However, our study has some limitations, including the fact that we are not able to guarantee that only women in the experimental group will use the chatbot-based decision aid; access to the chatbot will not require individual identification or codes. The chatbot will be accessible to everyone, which may bias the final results of our trial. This issue is common to all technology-based interventions, and it is not controllable. The AppDate-You Study Group decided not to evaluate some IPDAS effectiveness criteria, such as (1) “choice made” and (2) “decision-making process,” because these criteria have been widely assessed in several trial situations, which have shown that a decision aid is effective in increasing knowledge, accurate risk perception, and value-based informed choice, and in reducing decisional conflict [16].

Chatbot communication methods are growing rapidly in the health care field. To date, few studies have evaluated the effect of these methods in health care. This study will help inform policy makers and health care professionals.

## Appendix

Multimedia Appendix 1 Peer review report.

## Abbreviations

CC: cervical cancer
CCS: cervical cancer screening
CRCDC-OC: Regional Cancer Screening Coordinating Centre of Occitanie
cRCT: cluster randomized controlled trial
HPV: human papillomavirus
HPVss: human papillomavirus self-sampling
IARC: International Agency for Research on Cancer
IPDAS: International Patient Decision Aid Standards
IRIS: Ilots Regroupés pour l’Information Statistique
SNDS: Système National des Données de Santé
SPIRIT-PRO: Standard Protocol Items: Recommendations for Interventional Trials–Patient-Reported Outcomes

## Data Availability

The data that support the findings of this study are available on request from the corresponding author. The data are not publicly available due to privacy or ethical restrictions.
