# Supplementary material for: Effectiveness of Artificial Intelligence–Assisted Decision-making to Improve Vulnerable Women’s Participation in Cervical Cancer Screening in France: Protocol for a Cluster Randomized Controlled Trial (AppDate-You)
Source: JMIR Res Protoc. 2022 Aug 2;11(8):e39288. doi: 10.2196/39288 (PMC9382552; doi:10.2196/39288)
Supplement: Multimedia Appendix 1 [file resprot_v11i8e39288_app1.pdf]

INSTITUT NATIONAL DU CANCER **PROJET RISP-21-009**

Reporting projet

N° de dossier :	RISP-21-009
Titre du projet :	L'aide de l'intelligence artificielle à la prise de décision pour améliorer la participation des femmes au dépistage du cancer du col de l'utérus en Région Occitanie-France - Artificial intelligence-assisted decision-making to improve women's participation to cervical cancer screening in Occitanie Region-France
Coordonnateur de projet :	Farida SELMOUNI

Rapporteurs

Rapporteur 1

Points forts.

The proposal is well written and well structured. The background is very detailed and the rationale for the study was presented. The intervention is clearly described with strong process for developing/testing the intervention materials.

Points faibles.

The decision to use a 'cluster' RCT was not fully explained.

Conclusions.

Strong proposal worthy of funding.

Rapporteur 2

Points forts.

The proposal is clearly written and well thought through. There is an innovative approach applying use of chatbots to cervical screening. Improving access to cervical screening for deprived women is relevant to the 2014-2019 cancer plan and 2018-2022 National Health Strategy. There is a strong rationale based on the existing literature. The intervention seeks not only to improve screening uptake but also improve informed decision making. It is future-orientated – embracing technology and benefits from being multi-language and designed for women with lower education attainment. It is excellent that the information will be provided in text or spoken language to further address literacy barriers. It is carried by a strong team of national and international researchers. The description of outcome measures is clear. The project draws on self care framework and Ottawa Decision Support Framework. It incorporates health economics.

Points faibles.

The aim is to increase HPV self-samples – which is a behaviour – however, a behavioural scientist does not currently appear to be included in the research team.

Conclusions.

I strongly support this application. It is innovative, addresses inequalities in cervical screening uptake, supports informed decision-making and have the potential to overcome literacy and language barriers which are significant issues in cancer screening.

Rapporteur 3

Points forts.

This is an innovative project that will develop and evaluate a digital decision aid involving a chatbot delivered via multiple media to try to increase women's response to vaginal self-sampling in HPV testing (HPVss). The focus on women of lower socio-economic status is a particular strength of the project. The project has been well thought through and represents a coherent programme of research. Collaborators have been well chosen, and the project seems highly feasible. The project incorporates an economic evaluation.

Points faibles.

The justification for using a decision aid to try to increase women's response to vaginal self-sampling (HPVss) could be strengthened. Although much is known about the barriers to cervical screening uptake, we know much less about the barriers to HPVss and, in particular, why less than 20% of women in two French studies returned a sample when a self-sampling kit was sent to their home (as stated on page 18 of the project description).The application states that the reasons will be clarified by an ongoing qualitative study. However, unless we understand what the barriers are, it is difficult to make a strong case for using a decision aid. If some women do not regard cervical screening as important or have other things to worry about, they may not use a decision aid.So it is important to ensure that the proposed intervention (decision aid) does actually address the key barriers. Related to the above point, the development of the decision aid needs to be informed closely by the views of the target population i.e. women of low socio-economic status who have not responded to a previous invitation for cervical screening. This is crucial. The views of the

target population are more important than those of clinicians in this respect. Although the project description implies that the views of the target population will be taken into account, it is not clear that they will be given the highest priority.

Conclusions.

Excellent project.

Comité d'évaluation

Commentaires du Comité.

The committee states it is a well thought, innovative and future orientated project, promising to contribute to the reduction of literacy barriers.

Date du comité.

29/10/2020

Avis du comité.

Projet financé
